# Hydrogen-based industry: a prospective transition pathway toward a low-carbon future

**DOI:** 10.1093/nsr/nwad091

**Published:** 2023-04-05

**Authors:** Yunlv Cheng, Renyang Zheng, Zhicheng Liu, Zaiku Xie

**Affiliations:** China Petroleum & Chemical Corporation, China; China Petroleum & Chemical Corporation, China; State Key Laboratory of Green Chemical Engineering and Industrial Catalysis, SINOPEC Shanghai Research Institute of Petrochemical Technology, China; China Petroleum & Chemical Corporation, China; State Key Laboratory of Green Chemical Engineering and Industrial Catalysis, SINOPEC Shanghai Research Institute of Petrochemical Technology, China

## Abstract

The hydrogen-based industrial systems are key enablers that can help save fossil energy, reduce pollution, and achieve high-quality development goals for the process industry in the future.

## IMPORTANCE OF THE HYDROGEN-BASED INDUSTRY

The process industry, a vital part of the national economy, is currently being challenged by excessive energy consumption and high CO_2_ emissions. It is time to promote the transition to a greener and more sustainable pathway. Hydrogen, a widely used raw material and flexible energy carrier, is expected to speed up the decarbonization of industry and transportation sectors. Thus, building a low-carbon industrial system based on hydrogen (especially green hydrogen) in the process industry is a promising way to save fossil fuel energy, reduce pollution, and achieve the goal of high-quality development.

**Figure 1.**
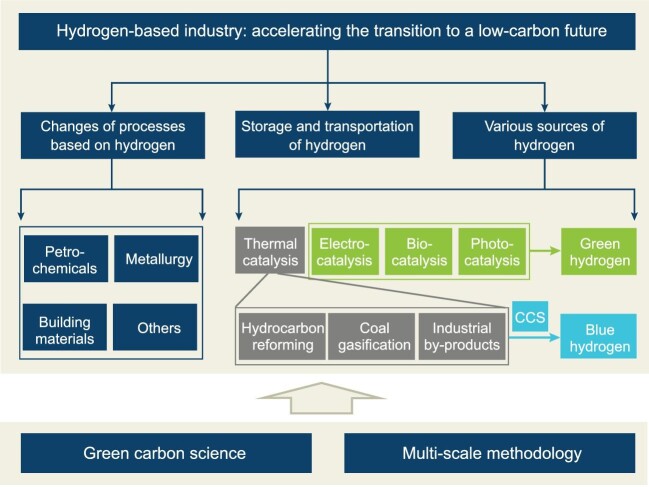
Schematic diagram of the hydrogen-based industry. There are three significant issues of the hydrogen-based industry: the changes of processes based on hydrogen, the storage and transportation of hydrogen, and the various sources of hydrogen, especially green hydrogen.

In order to establish the hydrogen-based industrial system, three significant issues have to be resolved: the changes of processes based on hydrogen, the storage and transportation of hydrogen, and the various sources of green hydrogen (Fig. 1). The concepts of green carbon science [1], as well as multi-level and multi-scale methodology [2], have provided critical scientific and technological supports.

## THE CHANGES OF PROCESSES BASED ON HYDROGEN

The changes of processes based on hydrogen refer to the re-design and re-distribution of energy and mass flows by integrating hydrogen into existing industrial processes. The integration with green hydrogen is effective in reducing the carbon footprint by shifting energy consumption from fossil fuels to renewable energy and simultaneously improving the resource utilization efficiency by maximizing the yield of desired products. It provides a long-awaited solution for hard-to-abate sectors including petrochemicals, metallurgy, and building materials.

### Petrochemicals

Hydrogen has been extensively used in oil refining such as hydrocracking and hydrofining, as well as in chemical processes involving ammonia and methanol synthesis. It is notable that substantial technical accumulations and theoretical breakthroughs have been made in the manufacture of high-end petrochemicals and in chemical reaction engineering. Looking ahead, it is crucial to follow the strategy of ‘precise catalysis’ [3], and to build a novel green–hydrogen–based refining and chemical system with shorter processes and less hydrogen consumption. Accordingly, many forward-looking technologies, such as efficient rearrangement and conversion of hydrocarbon molecules, direct cracking of crude oils to olefins, and methane dry reforming with CO_2_ to syngas, are calling for further research.

### Metallurgy

Iron and steel production emits a large amount of CO_2_, mainly because of the heavy dependence on coal as both the reductant and the thermal source [4]. A preferred option for cutting emissions is to substitute carbon with green hydrogen. Indeed, frontier research on hydrogen-rich blast furnaces and direct reduction in gas-based shaft furnaces is well under way. The key is to improve the degree of reduction and metallization, as well as to remove impurities in the refining processes.

### Building materials

It has been demonstrated that the production of cement (clinker) accounts for a significant share of greenhouse gas (GHG) emissions in the building materials sector [5]. Calcination reactions of the raw materials such as limestone is the dominant contributor. To this end, it is urgent to explore the mechanism of carbonate decomposition coupled with *in-situ* reduction, design practicable hydrogen donors, and create intrinsically safe models. These methods will contribute to the conversion of carbon species with high selectivity and high added values at lower temperature. Another main source of GHG emissions in the building materials sector is the combustion of coal-based fuels. Both switching to alternative fuels and promoting *in-situ* capture and conversion of GHGs in kiln flue gas will eliminate it.

## THE STORAGE AND TRANSPORTATION OF HYDROGEN

Hydrogen storage and transportation technologies are crucial to the development of hydrogen-based industry. As an energy carrier capable of long-term and large-scale storage, hydrogen is supposed to solve the space-time mismatch between demand and supply of renewable energy. At present, compressed storage and pipeline transport are feasible options with several problems including low cost-effectiveness and causing embrittlement. In the future, it seems more promising to store and transport hydrogen in forms of liquid chemicals such as methanol, ammonia, or organic liquids (e.g. methylcyclohexane) [6]. Admittedly, it requires tremendous efforts to develop direct catalytic hydrogenation of CO_2_ to liquid fuels and chemicals with high efficiency and high selectivity, or explore electrochemical ammonia synthesis with an industrial-scale yield.

## THE VARIOUS SOURCES OF GREEN HYDROGEN

Currently, hydrogen is mostly generated from hydrocarbon reforming, coal gasification, and industrial by-products, requiring carbon capture and storage (CCS) technologies to reduce emissions. It is attractive to produce hydrogen through low-carbon pathways using clean energy sources such as nuclear, wind, solar, geothermal, and hydroelectric power. Technology innovations in thermal catalysis, electrocatalysis, biocatalysis, and photocatalysis will facilitate these processes. Among various hydrogen production technologies, splitting of water using renewable electricity is recognized as a zero-emission and pollution-free route, offering an opportunity to produce H_2_ in a green manner.

### Alkaline water electrolysis (AWE)

AWE is a mature and reliable commercial technology, and has already made impressive strides over the past few years. For future adaptation to increasing demands, it is urgent to address some issues including low operational current density and limited flexibility [7].

### Proton exchange membrane water electrolysis (PEMWE)

PEMWE can operate at a higher cell pressure with a higher current density compared with AWE [8]. It is especially suitable for the local consumption and independent peak regulation of off-grid power with the advantage of quick response to fluctuation. Further studies should be centered on the development of the catalysts with high selectivity and low content of noble metals at the industrial-scale current density, the production of core components (e.g. bipolar plates) with high conductivity and high corrosion resistance, and the assembly of the electrolyzers that match industrial needs.

### Anion exchange membrane water electrolysis (AEMWE)

The emerging AEMWE deserves more attention because it can produce hydrogen at a competitive price [9]. Currently, numerous state-of-the-art technologies are in development, including self-supporting non-precious metal electrode, cross-linked or composite anion exchange membrane, as well as 3D-ordered membrane electrode assembly.

### Solid oxide electrolytic cell (SOEC)

SOEC is an advanced technology with high efficiency for hydrogen production. Furthermore, it can produce syngas via co-electrolysis of CO_2_ and H_2_O, paving a new way for CO_2_ utilization [10]. Considering the barriers for its scaling-up deployment lying ahead, significant advancements in core components are essential to improve the stability and durability at high operation temperatures.

## PERSPECTIVE

According to the International Energy Agency (IEA), by 2050, hydrogen will become an important pillar of industrial decarbonization, and will account for a tenth of global energy consumption [11]. While the establishment of an industrial system based on hydrogen is important and promising, there are many challenges in large-scale green hydrogen production, storage, transportation, and efficient utilization. Thus, it is worth carrying out target-oriented fundamental research, laying out transformative technologies in advance, and allocating more resources to key areas and weak links. It is certain that the establishment of the hydrogen-based industrial system will accelerate the transition of the process industry towards a low-carbon future.

## Supplementary Material

nwad091_Supplemental_File
