# Supplementary material for: Hydrogen-based industry: a prospective transition pathway toward a low-carbon future
Source: Natl Sci Rev. 2023 Apr 5;10(9):nwad091. doi: 10.1093/nsr/nwad091 (PMC10411679; doi:10.1093/nsr/nwad091)
Supplement: nwad091_Supplemental_File [file nwad091_supplemental_file.docx]

The hydrogen-based industrial systems are key enablers that can help save fossil energy, reduce pollution, and achieve high-quality development goals for the process industry in the future.
